# Year-round effects of a four-week randomized controlled trial using different types of feedback on employees’ physical activity

**DOI:** 10.1186/s12889-018-5402-0

**Published:** 2018-04-12

**Authors:** Karen Van Hoye, Anne I. Wijtzes, Johan Lefevre, Stijn De Baere, Filip Boen

**Affiliations:** 1 Department of Movement Sciences, Physical Activity, Sports & Health Research Group, KU Leuven, Tervuursevest 101, 3001 Leuven, Belgium; 2 Fontys University of Applied Sciences, KU Leuven, Tervuursevest 101, 3001 Leuven, Belgium

## Abstract

**Background:**

This follow-up study investigated the year-round effects of a four-week randomized controlled trial using different types of feedback on employees’ physical activity, including a need-supportive coach intervention.

**Methods:**

Participants (*n* = 227) were randomly assigned to a Minimal Intervention Group (MIG; no feedback), a Pedometer Group (PG; feedback on daily steps only), a Display Group (DG; feedback on daily steps, on daily moderate-to-vigorous physical activity [MVPA] and on total energy expenditure [EE]), or a Coaching Group (CoachG; same as DG with need supportive coaching). Daily physical activity level (PAL; Metabolic Equivalent of Task [MET]), number of daily steps, daily minutes of moderate to vigorous physical activity (MVPA), active daily EE (EE > 3 METs) and total daily EE were measured at five time points: before the start of the 4-week intervention, one week after the intervention, and 3, 6, and 12 months after the intervention.

**Results:**

For minutes of MVPA, MIG showed higher mean change scores compared with the DG. For steps and daily minutes of MVPA, significantly lower mean change scores emerged for MIG compared with the PG. Participants of the CoachG showed significantly higher change scores in PAL, steps, minutes of MVPA, active EE, total EE compared with the MIG. As hypothesized, participants of the CoachG had significantly higher mean change scores in PAL and total EE compared with groups that only received feedback. However, no significant differences were found for steps, minutes of MVPA and active EE between CoachG and PG.

**Conclusions:**

Receiving additional need-supportive coaching resulted in a higher PAL and active EE compared with measurement (display) feedback only. These findings suggest to combine feedback on physical activity with personal coaching in order to facilitate long-term behavioral change. When it comes to increasing steps, minutes of MVPA or active EE, a pedometer constitutes a sufficient tool.

**Trial registration:**

Clinical Trails.gov NCT01432327. Date registered: 12 September 2011.

## Background

The positive health outcomes of regular physical activity (PA) have been well documented [1]. However, despite the irrefutable evidence, a majority of the adult population worldwide does not reach recommended levels of PA [2, 3]. Different guidelines exist such as the 30 min of moderate to vigorous activity per day [4] or the guideline of 10,000 steps per day, which is more familiar to the media and general public [5]. These guidelines focus towards improving overall health and reducing the risk of several chronic diseases. However, these activity levels might be insufficient to maintain a healthy body weight. Several organizations have declared that adults should attain a physical activity level (PAL) of 1.75 or more to prevent excessive weight gain and avoid the transition to overweight or obesity [6]. In an attempt to promote and maintain sufficient levels of PA, a variety of methods have been developed, including behavioral interventions that focus on self-monitoring and/or need-supportive coaching [7]. The self-monitoring of PA, i.e. the daily recording of activity to track change, assists individuals in raising awareness of their current behavior and evaluating their performance in relation to specific goals/benchmarks such as current PA guidelines [8].

So far, individually tailored exercise strategies and feedback interventions have shown promising results [9, 10]. In order to design interventions to promote changes in health behaviors, more and more studies have been inspired by the Self-Determination Theory [11]. One construct from this theory, need support, has been shown to be especially promising in promoting long-term adherence to a physically active lifestyle [12]. Need-supportive coaching consists of a structured and individualized process of assistance in behavior change. This type of coaching should include support for three basic needs: autonomy (i.e. making your own choices), competence (i.e. to feel effective and confident in your own abilities and actions) and relatedness (i.e. to feel a sense of meaningful and mutual connectedness with others) [11].

A review on lifestyle physical activity interventions showed that those using self-monitoring and goal-setting are effective at increasing and maintaining levels of PA [13]. However, the majority of previous studies have been short-term in nature (1–15 weeks) [14]. One of the major questions concerning feedback methods to increase levels of PA is whether any positive changes are sustained over the long term [15], a prerequisite for achieving related health benefits [16].

Based on the protocol paper [17], the present manuscript describes the results of different feedback interventions from baseline to three follow-up time points, namely at 3 months, 6 months and 12 months. The results of the interventions from baseline to post-test have been published before [18] and demonstrated the short-term effects of different degrees of feedback. More specifically, participants receiving need-supportive coaching in addition to display feedback showed significantly higher PA levels post intervention compared with the display group [18]. However, no significant differences emerged between a no-feedback group and a pedometer only feedback group, nor between a pedometer only group and a display group (information on both PA and energy expenditure) post intervention.

Because behavioral change process may take longer than four weeks to become visible, the aim of the present study is to examine the sustainability of patterns of PA following a 4-week intervention using different types of feedback, including a need-supportive coaching intervention. We hypothesized that an intervention using feedback would result in a higher physical activity level one year later compared with the control group. Furthermore, looking at the pre-post intervention results that were previously published [18], we predicted that feedback combined with need-supportive coaching sessions would result in a lower decline in PA over a one-year follow-up period compared with participants only receiving continuous self-monitoring. In addition, we wanted to assess the change in PA patterns over time by including not only baseline, post-intervention and long-term (i.e. 12-month follow-up) measurements but also measurements at 3 month and 6 month follow-up.

## Methods

### Power calculation

Power calculations were performed using the results of a non-published pilot study (*n* = 73). In this pilot study, data were obtained on Physical Activity Level (PAL) using the SenseWear Armband (SWA). Conventional levels of statistical power (0.8) and level of significance (0.05) were used in the two-sided test. The pilot study involved two groups: an intervention arm receiving feedback on their physical activity level and a control group receiving no feedback. This pilot study revealed an estimated an effect size of 0.38, which corresponds to a mean difference in PAL of 0.10 METs. These calculations showed that a minimum of 57 participants per intervention arm was required for our randomized controlled trial. Power analyses were performed using statistical software G*Power (Heinrich-Heine-Universität, Düsseldorf).

### Study design

The study methods have been described in detail elsewhere [17]. In summary, after baseline measurement, participants having a PAL lower than 1.71 METs were randomly allocated to one of four groups: the Minimal Intervention Group (MIG) who received no feedback during the intervention, the Pedometer group (PG) who received continuous feedback on steps by a pedometer, the Display Group (DG) who received continuous feedback on steps, moderate to vigorous physical activity (MVPA), and energy expenditure (EE) by a SWA, or a Coaching Group (CoachG) who were supported by a personal coach in addition to continuous feedback by a SWA. The participants received different types of feedback according to their group allocation during a 4-week period. After the intervention period, participants were asked to turn in their feedback device. For randomization, participants were blindfolded and asked to choose a card from a deck of playing cards, with each symbol (clubs, diamonds, hearts and spades) representing a different intervention arm. Physical activity variables were assessed at baseline, 1 week after the 4-week intervention (post), at 3-month, 6-month, and 1-year follow-up using a SWA without feedback for a measurement period of 1 week. Our primary outcome variable included daily PAL (METs). Secondary outcomes included the number of daily steps, daily minutes of MVPA (defined as PA > 3 Metabolic Equivalents of Task [METs]), daily active EE (EE for all activities > 3 METs; kcal) and daily total EE (kcal).

Recruitment and baseline measurements took place between July 2010 and July 2011 with follow-up measurements collected between October 2010 and July 2012. Full ethical approval for this study was obtained by the Medical Ethics Committee of the KU Leuven. Each participant signed an informed consent. The trial is registered with ClinicalTrials.gov, number NCT01432327.

### Study population

Potential participants were recruited through local pharmacies and doctor practices and invited to an information session. Only those who reported not to be physically active were eligible for baseline measurements. At baseline, information on demographic characteristics, medical history, job status, sports and activity history, and physical (in)activity was collected by questionnaires, including the Flemish Physical Activity Computerized Questionnaire (FPACQ; [19]). Furthermore, participants underwent anthropometric and body composition measurements performed by trained staff following standard operating procedures and were fitted with a SWA, which they were asked to wear for a period of 7 days and nights.

**Fig. 1:**
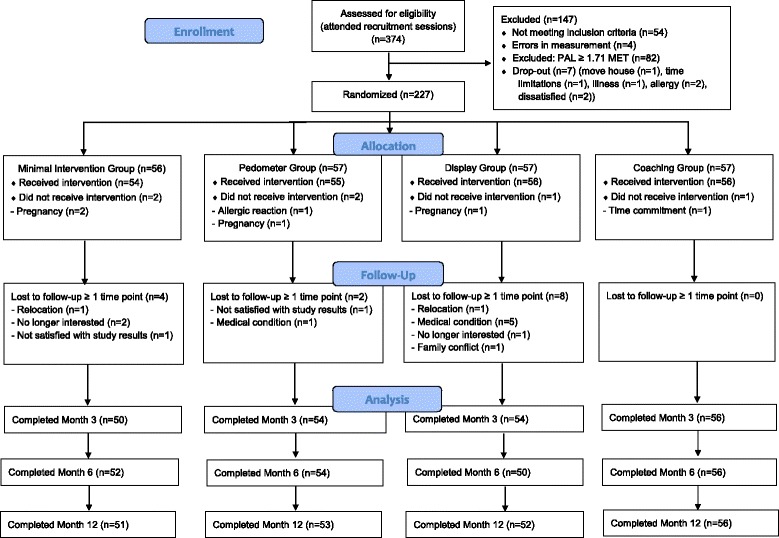
Participant flow diagram. PAL = physical activity level

As determined by our pilot study, individuals with PAL < 1.71 MET assessed by the SWA, were randomly allocated to one of the four study arms. In total, 103 male participants and 124 female participants aged 19–67 years were included in the randomization process. Drop-out percentages 1 year after randomization were 5.6%, 3.6%, 7.1% and 0% for the MIG, PG, DG and CoachG, respectively. Taking into account non-completers (e.g., participants who missed one or more assessments), objective data from 207 participants (91.2%) were obtained at baseline, after the intervention and at post 3 months, post 6 months and post 1 year with 50 participants in the MIG, 53 participants in the PG, 48 participants in the DG and 56 participants in the CoachG. Non-completers were not significantly different from completers for the measured anthropometric variables and the physical activity variables at baseline (results not shown). A flowchart of the recruitment and randomization processes is presented in Fig. 1.

### Intervention arms

At baseline, a personalized physical activity report was orally presented to all participants including baseline information on objectively measured daily PAL, daily steps, daily MVPA, daily active EE and daily total EE compared with the current PA recommendations as proposed by the American College of Sports Medicine [20]. Furthermore, participants were given information on the EE of familiar activities (e.g. housework, walking, cycling) so that they knew how much they needed to move to reach their personalized activity goals (i.e. change in PAL ≥0.1 MET).

MIG participants only received this report and did not receive any feedback during the four-week intervention. The PG received feedback on their number of steps from a Yamax SW Digiwalker, which is identified as one of the most accurate and reliable electronic pedometers available [21]. The DG and CoachG received feedback on daily steps, daily minutes of MVPA and total daily EE in real-time from a SWA display. Because there is evidence that goal setting is associated with positive behavior change [22], specific and individualized goals were also put into the SWA display.

During the 4-week intervention, each participant of CoachG had one face-to-face meeting of 30 min with a personal coach each week. During these meetings they discussed their physical activity behavior by means of a series of line graphs that displayed the daily step count, daily minutes of MVPA, and active daily EE and total daily EE. The personalized feedback was based on concepts of the SDT of Deci and Ryan [11]. The coach used need-supportive strategies and provided participants with choice (“What kind of activities would you like to do during lunch break?”), opportunities for initiative-taking (“Which type of exercises have you done during the past week that was fun?”), and constructive feedback (“You really did a nice job spending more time at physical activity during the weekend. Maybe now you can try to do this also on a workday? You will see that by doing so you will have more energy managing other tasks during the week”). In order to evaluate the quality of the need-supportive coaching, we asked participants of all groups to fill out an evaluation form after completing the 4-week intervention period. Because the participants of the MIG, PG and DG had one meeting with the coach after completing the baseline measurement to discuss their physical activity data, we were also interested in the opinion of those participants on evaluating this coaching session. The items included in this evaluation concerned the fulfillment of the need for autonomy, competence and relatedness [11] and were rated on a 5-point Likert scale (1 = strongly disagree, 2 = disagree, 3 = don’t know, 4 = agree and 5 = strongly agree).

### Physical activity data collection and processing

Objectively measured physical activity was assessed at baseline and at all follow-up measurements using the SWA, a multisensory activity monitor that incorporates different heat related sensors and a 2-dimensional accelerometer. The device records daily step count, daily active EE and total daily EE, the intensity and duration of physical activity bouts, and sleep efficiency. The device is non-invasive and is worn on the upper right arm. The SWA itself has no display function but can be linked to a SWA display. Consequently, individuals that only wear the SWA do not receive any information on their physical activity behavior that could alter their activity level. One reason why an intervention period of 4 weeks was chosen is because participants had to wear the SWA device during waking hours. This device was rather large and uncomfortable. We therefore decided to limit the intervention to 4 weeks, also because previous intervention of that duration had proven to be successful.

The SWA is proven to be valid for light and moderate intensity activities [23] but tends to underestimate higher intensity activities [24, 25]. The monitor was set to record at 1-min epochs. At baseline and during follow-up measurements, participants were asked to wear the SWA all day and night for seven consecutive days (24/7). The SWA was used to objectively monitor physical activity behavior without providing any activity feedback to the participant. Participants were asked to remove the SWA only for water-based activities such as bathing, showering and swimming. Missing values due to water-based activities were imputed with the corresponding energy values according to the table of Ainsworth et al. [26]. Each day with more than 5% of missing data (equivalent to 72 min of no data) was excluded from the analysis and average physical activity variables were then estimated based on the remaining days. Participant’s data were used only when there was valid data for at least three weekdays and both weekend days [27]. SWA data cleaning was performed using SAS 9.2 (SAS Institute, Cary, NC, USA).

### Statistical analysis

Descriptive baseline characteristics of the four intervention arms are tabulated as means and standard deviation scores (SDs) or as percentages. Differences between the groups were analyzed using ANOVA for continuous variables and Chi-square tests for categorical variables.

Differences between the four study arms in PA variables were tested consistent with the intention-to-treat approach [28], where study participants are analyzed as members of the treatment group to which they were randomized regardless of their adherence to the intended treatment. For example, when participants could not be monitored at 3 months they were allowed to continue their participation at the trial at 6 months or 1 year. PA variables were inspected for normality using a Shapiro-Wilk test. Mean change scores were calculated for the different time points (post intervention, post 3 months, post 6 months and post 1 year) and for the different PA variables (daily PAL, number of daily steps, daily minutes of MVPA, active daily EE and total daily EE). Differences in change scores in normally distributed PA variables were analyzed using a two-way repeated measures ANOVA with Group and Time as independent variable. Differences in change scores in skewed PA variables were analyzed using Kruskal Wallis tests by ranks (main effect for Group), Friedman aligned rank tests (main effect for Time), and aligned rank test for interaction (interaction Group*Time). Post-hoc multiple comparison analyses focused on comparing change scores between those receiving no feedback (MIG) to all others and those receiving feedback (PG and DG) to those receiving feedback and coaching (CoachG). The Duncan’s new multiple range test was used in normally distributed PA variables, the Dunn’s rank test in skewed PA variables.

To assess any differences in the quality of need-supportive coaching between the different intervention arms, Kruskal-Wallis tests were used. Analyses were performed using SAS 9.2 and R v1.5 statistical software, applying a significance level of *p* <  0.05.

## Results

### Characteristics of the study population

Mean wear time of the armband at baseline, one week after the intervention and at post 3 months, post 6 months and post 1 year was calculated for the total group of participants. At baseline, following data cleaning, participants wore the device on average 98.5 ± 2.8% of the day, which corresponds to a wear time of 1418 min or 23.6 h. Participants had an average wear time of 93.5 ± 12.5%, 98.0 ± 3.0%, 98.1 ± 3.2% and 98.0 ± 3.3% at post, post 3 months, post 6 months and post 1 year, respectively. This corresponds to an average wear time of 1350 min or 22.5 h per day at post and 1412 min or 23.5 h per day at the other follow-up measurements.

Baseline participant characteristics are presented in Table 1. Participants did not differ significantly with respect to sociodemographic, biological and behavioral characteristics. Significant differences were observed for daily PAL and daily steps. Despite randomization, the DG had a significantly higher daily PAL compared with the PG (0.08 ± 0.03 MET, *p* <  0.05), which translates into an increased EE of 117 kcal per day for an individual with a body weight of 60 kg. Furthermore the participants in the DG also took more daily steps at baseline compared with the PG (mean diff: 2239 ± 559 steps, *p* <  0.001). No baseline differences were found for active daily EE, total daily EE, or daily minutes of MVPA. Because of these baseline differences between groups, all analyses were performed with mean change scores which took into account the baseline values of the physical activity variables.

**Table 1:** Baseline participant characteristics (*n* = 227)

Participant characteristics	MIG (*n* = 54)	PG (*n* = 55)	DG (*n* = 56)	CoachG (*n* = 56)	*p*-Value
Gender (male)	46	46	46	45	0.997
Marital status (currently married)	42	64	60	46	0.529
Education (higher educated)	67	71	61	71	0.990
Smoking status (smokers)	15	7	7	11	0.557
Age (years)	41.2 ± 11.0	43.3 ± 10.7	44.3 ± 9.9	40.7 ± 9.8	0.211
BMI (kg/m^2^)	26.4 ± 3.3	26.8 ± 4.2	27.5 ± 3.9	27.8 ± 4.5	0.216
Body fat (%)	28.4 ± 6.6	29.4 ± 6.7	29.6 ± 6.9	29.4 ± 5.8	0.753
SBP (mmHg)	122.7 ± 16.0	126.7 ± 17.4	125.7 ± 16.4	120.8 ± 15.0	0.193
DBP (mmHg)	82.3 ± 10.3	83.2 ± 11.4	83.3 ± 10.4	78.6 ± 8.5	0.056
FPACQ (METs)	1.68 ± 0.20	1.63 ± 0.11	1.71 ± 0.19	1.68 ± 0.16	0.148
Daily steps (number)	9855 ± 2983	8840 ± 2306	11,079 ± 3431	9978 ± 2940	0.001
Daily MVPA (minutes)	116 ± 42	101 ± 45	118 ± 46	107 ± 51	0.186
Active daily EE (kcal)	595 ± 234	516 ± 245	638 ± 305	578 ± 289	0.125
Total daily EE (kcal)	2713 ± 402	2634 ± 484	2835 ± 533	2751 ± 534	0.187
PAL (METs)	1.46 ± 0.14	1.39 ± 0.16	1.47 ± 0.16	1.41 ± 0.17	0.030

*MIG* Minimal Intervention Group, *PG* Pedometer Group, *DG* Display Group, *CoachG* Coaching Group, *BMI* body mass index, *SBP* systolic blood pressure, *DBP* diastolic blood pressure, *FPACQ* Flemish Physical Activity Computerized Questionnaire, *METs* metabolic equivalent of task, *MVPA* moderate-to-vigorous physical activity, *EE* energy expenditure, *kcal* kilocalories, *PAL* physical activity level

Values are means and standard deviations (SD) for continuous variables and percentages within group for categorical variables. Differences between groups were tested with ANOVA for continuous variables and Chi-square for categorical variables

### Intervention effectiveness

To investigate whether participants of the different groups increased their activity level at the different time points we expressed their change as relative scores (Table 2). Means (percentages) and standard deviations are presented for the relative change in PAL, steps, minutes of PA, active EE and total EE measures at respectively post, post 3 months, post 6 months and post 1 year for the different intervention arms. The effect outcomes of the two-factor mixed design for the different physical activity variables are presented in Table 3.

**Table 2:** Relative change scores (%) at the various time points for all intervention arms (*n* = 207)

		Post	Post3m	Post6m	Post1y	Group effect^*^
PAL (METS)	MIG	0.6 ± 7.0	3.0 ± 8.4	0.6 ± 7.4	1.5 ± 8.4	abab
	PG	0.7 ± 8.1	1.8 ± 9.9	2.4 ± 7.9	1.1 ± 7.0	b
	DG	0.1 ± 8.6	− 1.0 ± 7.5	0.9 ± 8.7	−1.5 ± 7.9	a
	CoachG	4.2 ± 7.7	6.0 ± 8.1	4.9 ± 9.4	2.5 ± 8.2	c
Daily MVPA (min)	MIG	9.6 ± 34.0	18.0 ± 50.4	9.3 ± 45.5	10.5 ± 42.0	ab
	PG	21.1 ± 65.1	28.2 ± 70.6	24.2 ± 58.9	17.4 ± 54.0	ac
	DG	8.4 ± 39.5	−1.7 ± 37.1	11.8 ± 46.0	−6.7 ± 33.9	b
	CoachG	22.9 ± 39.0	35.2 ± 56.0	29.4 ± 63.3	18.3 ± 56.0	c
Act. daily EE (min)	MIG	13.4 ± 39.1	19.6 ± 53.2	9.0 ± 49.8	11.9 ± 44.6	ab
	PG	26.1 ± 77.1	31.4 ± 74.5	26.8 ± 64.1	20.1 ± 58.5	ac
	DG	12.9 ± 44.5	1.0 ± 40.8	13.5 ± 47.2	− 3.3 ± 38.9	b
	CoachG	30.2 ± 50.6	38.3 ± 58.7	32.7 ± 66.5	22.7 ± 68.4	c
Total daily EE (min)	MIG	0.7 ± 7.0	1.6 ± 7.8	−0.5 ± 7.1	0.8 ± 7.8	ab
	PG	0.8 ± 8.0	1.9 ± 9.6	2.2 ± 8.0	2.0 ± 7.5	a
	DG	0.2 ± 8.7	−1.3 ± 8.6	0.4 ± 8.8	−1.5 ± 7.9	b
	CoachG	4.3 ± 7.7	5.3 ± 8.4	4.9 ± 8.7	3.0 ± 9.0	c
Daily steps (#)	MIG	6.7 ± 26.0	7.8 ± 24.4	2.8 ± 23.0	3.9 ± 22.6	a
	PG	13.1 ± 30.9	17.6 ± 33.4	12.7 ± 20.3	7.0 ± 21.5	b
	DG	4.4 ± 25.9	2.3 ± 21.4	2.5 ± 22.9	−0.2 ± 23.2	a
	CoachG	20.7 ± 29.9	18.9 ± 31.9	17.3 ± 27.3	13.6 ± 28.2	b

Values are relative change scores (means ± standard deviations) within group**s**. Post = difference between post and baseline data; Post3m = difference between post 3 months and baseline; post6m = difference between posttest 6 months and baseline; post1y = difference between posttest 1 year and baseline; *MIG* Minimal Intervention Group, *PG* Pedometer Group, *DG* Display Group, *CoachG* Coaching Group, *PAL* physical activity level, *MVPA* minutes of moderate to vigorous physical activity, Active daily EE = energy expenditure > 3 Mets; Total daily EE = total energy expenditure

*Post-hoc effects for between group differences: groups with the same letter are not significantly different across time points

**Table 3:** Effect outcomes of the two-factor mixed design for the different physical activity variables

PA variable	Effect	F/X^2^	*P*
PAL	Group ^a^	F = 6.02	< 0.001
	Time ^a^	F = 2.49	0.06
	Group*Time ^a^	F = 1.43	0.17
Daily MVPA	Group ^b^	X^2^ = 24.35	< 0.001
	Time ^c^	X^2^ = 829.45	< 0.001
	Group*Time ^d^	F = 1.43	0.17
Active daily EE	Group ^b^	X^2^ = 27.00	< 0.001
	Time ^c^	X^2^ = 829.27	< 0.001
	Group*Time ^d^	F = 1.89	0.05
Total daily EE	Group ^a^	F = 6.33	< 0.001
	Time ^a^	F = 0.91	0.43
	Group*Time ^a^	F = 1.37	0.20
Daily steps	Group ^b^	X^2^ = 52.45^b^	< 0.001
	Time ^c^	X^2^ = 830.16^b^	< 0.001
	Group*Time ^d^	F = 0.69 ^d^	0.72

*PAL* physical activity level, *MVPA* minutes of moderate to vigorous physical activity, Active daily EE = energy expenditure > 3 Mets; Total daily EE = total energy expenditure. ^a^Two-way repeated measures ANOVA; ^b^Kruskal-Wallis test by ranks; ^c^Friedman aligned rank test; ^d^aligned rank test for interaction

Our first aim was to compare those receiving no feedback (MIG) to all others. No significant Group*Time interaction effect were found for all variables. Main effects for Time emerged for daily steps, daily minutes of MVPA and active daily EE. Overall, physical activity levels were lower at 1 year follow-up compared to post-intervention despite the high values in the CoachG condition. Main effects for Groups emerged for all PA variables. Participants of the MIG had significantly lower change scores in PAL (1.4% ± 7.7), daily steps (4.9% ± 24.0), daily minutes of MVPA (12.6% ± 43.4), active daily EE (13.5% ± 46.0), total daily EE (0.58% ± 7.4) compared with the CoachG (resp. 4,4% ± 8.4; 17.6% ± 29.3; 26.5% ± 54.3; 31% ± 61.3; 4.4% ± 8.4). Only for daily minutes of MVPA higher mean change scores were reported for the MIG compared with the DG (3.8% ± 39.8). Significantly lower mean change scores were reported for MIG compared with the PG for daily steps (12.7% ± 26.9) and daily minutes of MVPA (22.2% ± 61.5).

Our second aim was to assess the comparative effectiveness of measurement feedback provided by a pedometer (PG) or a display (DG) versus receiving feedback *plus* coaching. A significant main effect for Group emerged for all variables. Post-hoc analyses showed that participants of the CoachG had significantly higher mean change scores in PAL (4.4% ± 8.4) and total daily EE (4.4% ± 8.4) compared with groups only receiving feedback (resp PG 1.7% ± 8.2 and 1.7% ± 8.3; DG -0.5% ± 8.2 and − 0.6% ± 8.5). No significant differences were found for daily steps, daily minutes of MVPA and active daily EE between CoachG and PG (resp. 17.6% ± 29.3 versus 12.7% ± 26.9; 26.5% ± 54.3 versus 22.2% ± 61.5; 31% ± 61.3 versus 26.3% ± 67.9) while there was a significant difference for these variables between CoachG and DG (resp. 17.6% ± 29.3 versus 2.0% ± 23.0; 26.5% ± 54.3 versus 3.8% ± 39.8; 31% ± 61.3 versus 5.5% ± 43.8).

### Responders versus non-responders

To assess the proportion of participants responding to the intervention at post-intervention and post 1 year, we categorized participants in three different groups: no effect (less active at post compared with baseline), relapse (more or equally active at post compared with baseline & less active at 1-year follow-up compared with post) or success (more or equally active at post compared with baseline & more or equally active at 1-year follow-up compared with post). Results showed that in general, 50% of participants of the MIG, PG, and DG did not change their physical activity behavior as a result of the intervention, whereas 25% of participants relapsed after an initial good effect and 25% successfully increased physical activity in the long-term. The distribution for the CoachG looked somewhat different, with about 40% of participants showing no effect of the intervention, 20% of participants relapsing after an initial good start, and 40% of participants showing positive long-term results.

### Quality of need-supportive coaching

Mean scores from the analyses of the questionnaire on need-supportive coaching showed that need-supportive coaching was rated higher by participants of the CoachG (4.54/5) compared with the DG (4.22/5), PG (4.21/5), and the MIG (3.91/5) (*p* = 0.013, *p* = 0.033, and *p* = 0.007, respectively). In addition, 90% of the participants of the CoachG (*n* = 50) stated that they knew what to do after the conversation with the coach (e.g. need for competence). More than half of them fully agreed with the statement that the coach also listened to their own ideas and that the coach taught them to find their own solutions for their problems (e.g. need for autonomy). Furthermore, 90% of participants (*n* = 50) indicated that they could depend on their coach, that there was a connection between the coach and them and that the coach was very empathically involved (e.g. need for relatedness).

## Discussion

This study evaluated the twelve-month effects of a four-week intervention using different types of feedback on employees’ physical activity levels, including a need-supportive coach intervention. Follow-up results were presented 1 week after the 4-week intervention period and 3 months, 6 months and 1 year after randomization.

In general, looking at the time effects independent of the group allocation, our study revealed a tendency for the change scores in minutes of MVPA, daily steps and active EE to drop after 1 year despite the high value in the coach condition. Other studies involving workplace samples have demonstrated complete regression to baseline values [29, 30]. These studies also use a short initial intervention (4-weeks). Studies show that PA behaviors are stable across long time periods, which suggests the difficulty of long-term PA behavior change sufficient for health benefits [31]. Despite the drop in relative change scores over time for all PA variables, the results of the responders versus non-responders of our study indicate that 40% of the participants of the CoachG showed positive long-term results.

The first aim of the present study was to compare those receiving no feedback versus those receiving feedback during the intervention by using a pedometer or a SWA display. Having a technological device giving real time feedback on steps, minutes of MVPA and total EE did not increase physical activity compared with receiving no feedback. Moreover, our results showed that participants receiving no feedback had a significantly higher increase in minutes of MVPA compared with those using the SWA display. On the other hand, participants using a pedometer showed a higher increase in relative steps and minutes of MVPA compared with those receiving no feedback. It thus seems that providing individuals with information on only one aspect of the behavior (e.g. steps) seems to have a more beneficial effect than providing information on multiple aspects of the behavior (e.g. steps and minutes of MVPA) and the behavioral outcome (e.g. total calories burned). This conclusion is in line with a study by Richardson et al. [32], which compared two goal-setting strategies including a strategy targeting total daily accumulated steps only and a physical activity program specifying a minimum duration and intensity of physical activity bouts. The authors determined which goal-setting strategy was more effective at increasing bout steps in sedentary adults with type 2 diabetes. All participants wore pedometers and received automated step-count feedback, automatically calculated goals, and tailored motivational messages throughout a six-week intervention. They found an increase in steps for both strategies at the end of the intervention (1921 ± 2729 steps) with no statistically significant differences between the groups. However, they indicated that participants who were given a daily step goal were more satisfied with and more adherent to the intervention compared with those who received structured goals. As the present study revealed, a greater adherence to a step goal by giving individuals information on only one aspect of the behavior (e.g. steps) could prevent a decrease in physical activity behavior in the long-term.

Participants of the MIG had significantly lower change scores in all PA variables compared with the CoachG. Our findings suggests that exposure to PA consultations in the intervention provided an advantage compared with those who only receive a brief counseling session with a coach after baseline measurement to discuss their PA report. Another study by Proper et al. [33] investigated the success of need-supportive coaching without using any self-monitoring device. A total of 299 employees of three municipal services in the Dutch town of Enschede were randomly allocated to an intervention (*n* = 131) and control group (*n* = 168). Over a 9-month period, subjects from the intervention group were offered seven counseling sessions. Counseling was based on the individual’s stage of behavioral change. Participants in both the intervention and control group received written information about several lifestyle variables. The authors found positive effects on total EE, percent body fat, and blood cholesterol compared to the control group [34]. The authors therefore recommend the implementation of PA counseling at the workplace to increase the proportion of employees who are physically active. Because our study did not include a coaching only group, it is difficult to predict if coaching only (without the measurement feedback) would have the same effects on the PA behavior. Our study does show however that having only one meeting with a coach after baseline measurement is not enough to sustain a long-term change in the PA pattern of individuals and that a weekly counseling session using subgoals and need-supportive feedback is recommended.

Our second aim was to assess the long-term effectiveness of feedback by a pedometer or SWA display versus feedback *plus* coaching. Providing coaching has been promoted as a useful adjunct to many health and well-being interventions. Our results showed higher mean change scores in PAL and total daily EE for participants of the CoachG compared with groups only receiving feedback. No significant differences were found however for daily steps, daily minutes of MVPA and active daily EE between CoachG and PG. Pedometers provide a simple, cost-effective means of motivating individuals to increase walking yet few studies have considered if short term changes in walking behaviour can be maintained in the long-term. Our study indicates that having the low-cost pedometer is of great value when it comes to increasing daily steps, minutes of MVPA and active daily EE. A similar study by Fitzsimons et al. [34] examined the effect of a 12-week physical activity counseling program in a pedometer-based intervention over a 1-year period. Ninety-seven low active Scottish men and women were randomly assigned to a pedometer-based walking program *plus* PA consultations or a pedometer-based walking program and minimal advice. Step counts were assessed pre-intervention and 12, 24 and 48 weeks after receiving the intervention. In line with our study results, both interventions successfully increased and maintained step counts over 12 months.

Several strengths and limitations of this study should be considered when interpreting the results. Retention rates over a 12-month period were much higher in this study compared with similar previous studies [33]. Several measures were taken to promote continued participation in the current study, including providing a personalized report after the intervention and 3 months, 6 months and 1 year after randomization, being flexible in meetings with participants, and appointment invitations by phone instead of by email. It should be noted that these follow-up measurements could have acted as boosters, which might explain the higher physical activity level of the participants of the CoachG even 1 year after the intervention. Other strengths of the present study include the large sample size and the high wear time of the SWA, therefore minimizing potential bias stemming from selective participation. Self-monitoring technologies such as the pedometer and the SWA come with several limitations in terms of user acceptance, facilitation of long-term commitment and suitability to different users, goals and activities [35]. However, according to our evaluation forms, only 4% of participants stated that the SWA armband was difficult to use.

A first limitation of this study is the fact that despite randomization, one group (i.e. the DG) was slightly more physically active at baseline compared to the PG. However, by using relative change scores, we took this baseline difference into account. Furthermore, participants were recruited through self-selection (volunteering) which may have led to a selected sample of highly motivated participants and thus potential limited generalizability of results to the general population. However, the results from the FPACQ administrated before baseline measurement showed that we had some success in reaching the inactive segment of the working population. More specifically, 7% of the participants indicated that they did not meet the physical activity recommendations and that they were not motivated to change this inactive behavior the following year. About 25% of the participants indicated that they did not meet the physical activity recommendations but that they wanted to change this behavior in the next 6 months. According to literature, these individuals are hard to reach and mostly do not respond to health promotion programs [36, 37]. Looking at the baseline data we should mention that the group of study was exceptionally active at baseline as all the groups at baseline (except for the participants of the pedometer group) were near the 10,000 steps per day recommendation. These high baseline scores question the generalizability of our findings. Study participants were included in the trial on their daily PAL and not on their daily step count. Future studies should take daily steps as an inclusion criterion instead of only considering the daily PAL. Finally, when considering the feasibility of conducting the present intervention on a larger scale, one should also take into account the cost-effectiveness of this type of intervention. Deliverance of motivational coaching, personalized feedback, and goal setting in face-to-face meetings with a coach is costly. However, these costs can be reduced by limiting the number of contacts with the coach [12]. Another potential strategy that could lower the cost of this type of intervention is the use of email-based feedback or the use of a web-based coach instead of face-to-face contacts. Nevertheless, the findings of a recent meta-review suggest that the potential of internet-delivered interventions to produce meaningful changes in long-term physical activity remains unclear [38].

### Perspectives

This randomized controlled trial adds to the existing literature on measurement feedback strategies by showing the potential of need-supportive coaching in addition to display feedback. Because more and more evidence indicates that people might be differentially influenced by intervention programs [37], it is important to identify subgroups of individuals who respond differently to interventions and to tailor future need-supportive interventions to the physical and psychological characteristics and needs of each subgroup.

## Conclusions

In conclusion, the current study demonstrates the potential importance of need-supportive coaching for long-term physical activity behavioral change. Based on our results, we propose that behavioral interventions can be accompanied by a personal coach to increase to their PAL and total daily EE. When it comes to increasing steps, minutes of MVPA or active EE, a pedometer constitutes a sufficient tool. Giving the evidence of the decline of physical activity scores after 1 year (despite the high values in the coach condition), it is important that individuals participating in such programs are provided with continuous feedback on their progress and are coached in a need-supportive climate.

## Abbreviations

CoachG: Coaching Group
DG: Display Group
EE: Energy Expenditure
FPACQ: Flemish Physical Activity Computerized Questionnaire
MET: Metabolic Equivalent of Task
MIG: Minimal Intervention Group
MVPA: Moderate-to-Vigorous Physical Activity
PA: Physical Activity
PAL: Physical Activity Level
PG: Pedometer Group
SD: Standard Deviation Score
SDT: Self-Determination Theory
SWA: SenseWear Armband
